# Analysis of equivalent uniform dose (EUD) and conventional radiation treatment parameters after primary and re-irradiation of malignant glioma

**DOI:** 10.1186/1748-717X-8-287

**Published:** 2013-12-13

**Authors:** Maximilian Niyazi, Ivan Karin, Matthias Söhn, Silke B Nachbichler, Peter Lang, Claus Belka, Ute Ganswindt

**Affiliations:** 1 Department of Radiation Oncology, University of Munich, Marchioninistr. 15, Munich 81377, Germany

**Keywords:** Re-irradiation, Malignant glioma, EUD, Radiation necrosis

## Abstract

**Background:**

Re-irradiation is a reasonable second treatment option for patients with recurrent malignant glioma (MG) after previous radio(chemo)therapy. However, only limited data is available allowing for a precise selection of patients suitable for re-treatment in regard to safety and efficacy.

**Methods:**

Using the department database, 58 patients with two courses of percutaneous radiation were identified. Besides classical dose-volume histogram (DVH) parameters equivalent uniform dose (EUD) values were calculated for the tumor and organs at risk (OARs), retrospectively analyzed and correlated to survival outcome parameters. Cumulative EUD values were also calculated in all cases where previous OAR DVHs were available.

**Results:**

Median follow-up was 265 days and no relevant toxicity was observed after re-irradiation in our patient cohort during follow-up. Time interval between first and second irradiation was regularly above 6 months. As a conservative estimation of the cumulative EUD to the OARs, the EUDs of first and second irradiation were added. Median cumulative EUD to the optic chiasm was 48.8 Gy (range, 2.5–76.5 Gy), 57.4 Gy (range, 2.7–75.3 Gy) to the brainstem, 20.9/22.1 Gy (range, 0.0–68.3 Gy) to the right/left optic nerve and 73.8 Gy (range, 64.9–77.3 Gy) to the brain. No correlation between treated volume and survival was seen.

**Conclusions:**

This study provides retrospective estimates on cumulative doses at the OARs. EUD values are derived and may serve as reference for further studies, including planning studies where specific constraints are needed.

## Introduction

The prognosis of patients with malignant glioma (WHO grades III + IV), and especially glioblastoma, is limited by a high rate of local failures [1-6]. Concurrent adjuvant radiochemotherapy with temozolomide (TMZ) has improved local control and survival [7,8]. However, 72.8% of the patients still die within 24 months [9].

In selected patients, a second course of radiotherapy is regarded as a reasonable re-treatment option [10-13]. The widespread availability of modern radiotherapy equipment [14-18], improved pre-treatment imaging capabilities and the fact that animal experiments in primates revealed a substantial repair of critical CNS structures [19] allowed the re-evaluation of this option in clinical practice [10,20,21].

At present, no clear guidelines exist when and for whom a second course of radiation may be performed. Additionally, there is no clear data on a preferential size for re-treatment volumes, optimal time interval between first and second irradiation (most authors demand an interval of at least six months [10,20,21]) and reliable dose volume constraints being predictive for relevant toxicity.

One previous analysis of our group focused on radiation treatment parameters of re-irradiation only not considering the pre-irradiation dose [22]; in order to estimate cumulative doses, one may focus on the so-called equivalent uniform dose (EUD) – a measure that can represent inhomogeneous dose distributions and the sum of different EUDs may serve as a conservative dose estimate – a clear advantage compared to peak doses.

Aim of this retrospective study was to collect different treatment parameters of primary and re-irradiation such as minimum, maximum, mean dose, cumulative dose estimates, treated volume as well as EUD, to correlate these parameters with survival, and to derive feasible dose constraints.

## Methods and materials

All patients treated with a series of re-irradiation for recurrent MG at the University hospital of Munich between 12/2006 and 3/2011 were identified using the department database. Altogether 58 patients were found who all had histologically and/or FET-PET/MRI proven recurrent malignant glioma, macroscopic tumor in the brain and available treatment plans. This study includes patients for whom treatment plans had been evaluated before [22].

### Treatment schedule and follow-up

Baseline evaluation included gadolinium-enhanced brain MRI with gradient echo sequence and perfusion. Treatment outcome as well as brain necrosis/leukoencephalopathy was evaluated on a regular basis by brain MRI or FET-PET and neurological status according to the RANO criteria [23]. Radiochemotherapy in the primary setting was (if chemotherapy was applied) in accordance with the respective EORTC trial [24]. The patient cohort consisted in part of previously reported patients [25] and patients who have been treated in the meantime with the same protocol. Median follow-up was 265 days.

### Radiotherapy

Patients underwent an MRI with ≤3 mm slices within two weeks of the treatment planning CT with 3-mm slices. Patients were immobilized with a thermoplastic mask system. PTV concepts from the first radiotherapy series were regularly in accordance with RTOG and EORTC standards. Dose (36 Gy, 2 Gy single fractions) and PTV concept (margin up to 10 mm) for re-irradiation has been employed as described before [25]. Treatment planning was performed employing the Helax® TMS 6.1B1 (Nucletron, Veenendaal, The Netherlands) or Oncentra® treatment planning system (OTP MasterPlan®, Nucletron, Veenendaal, The Netherlands). In order to achieve a uniform database and a consistent dose calculation, Helax® treatment plans were re-calculated using the OTP software package for further analysis. Thirty-one patients have initially been treated elsewhere and several treatment planning systems were employed for dose calculation with Helax® being the most frequent one (N = 20).

### EUD concept

The concept of equivalent uniform dose (EUD) assumes that different dose distributions are equivalent if they are able to elicit the same radiobiological effect. Thus EUDs are specific for a pre-defined and quantifiable biological endpoint. An EUD can be calculated directly from the dose calculation points or, from the corresponding dose-volume histograms (DVHs) [26].

Typically EUD is between the minimum and mean dose for tumors and between the mean and maximum dose for critical structures (especially serial organs). EUD can be a useful endpoint in evaluating treatment plans with non-uniform dose distributions for 3D conformal radiotherapy (3D-CRT) and intensity-modulated radiotherapy (IMRT) [27]. EUDs within this paper were calculated as described before [22]. In this study, empirical parameters were used to obtain corresponding EUDs (k = 5 for the brain, k = 12 for all other structures and α=0.4), for review see [28,29].

### Cumulative EUD and recovery

To encounter the problem of estimating the cumulative dose in OAR tissues with two sequential radiation treatments the relation was used that the sum of two EUD measures may be regarded as upper limit of the actual total EUD [30]. In a second step an empiric recovery factor of r = 0.5 for the primary radiation dose was introduced which should account for repair of normal CNS tissue; this uniform value was rather an approximation and simplification as e.g. the optical system and remaining CNS tissues certainly differ in their capacity of recovery. In this context, the corrected sum was stated as well as the total cumulative dose without any corrections.

### DVH analysis

For several treatment plans, no previous DVH raw data but hardcopies were available. In these cases, raw data were derived from the printed DVH curves using the open source digitizing program “Engauge Digitizer” (http://digitizer.sourceforge.net/).

### Statistics

Demographic data, volume and dose parameters as well as treatment response were analyzed using descriptive statistics; comparisons between groups/parameters were carried out using Fisher’s exact test/asymptotic χ^2^–tests, Mann–Whitney *U*-test or Wilcoxon test. Survival analyses and univariate analyses were based on Kaplan-Meier estimates. For all patients, survival was measured from the first day of re-irradiation until death or last follow-up. A two-tailed p-value ≤0.05 was considered significant.

## Results

### Patient characteristics

Altogether 58 patients were retrospectively analyzed (male:female ratio = 1.6:1). The median age of the patients was 52 years, 48.3% of the malignant glioma (MG) of this patient population were not methylated (in 17.2% of the cases the methylation status was unknown). Surgery had been performed in 47 patients (81%). Radiotherapy was performed with a median dose of 60 Gy as an adjuvant (after surgery) or primary therapy at first diagnosis (details are listed in Table 1). Eight patients received a brachytherapy (iodine seed implantation) between first and second percutaneous irradiation – the corresponding dose contribution was not considered in the further analysis; calculations were also performed disregarding these patients but are not presented due to clarity reasons and lack of additional information. The main planning system for the first course of radiation was Helax TPS with 34.5%; the main planning system at the time of re-irradiation was Oncentra® Masterplan (72.4%). Only 27 (46.6%) of the patients were treated at the University hospital of Munich for the first and second radiation course.

**Table 1:** Patient characteristics

**Characteristic**	**Patients (N = 58)**
**Sex**	
**• Male**	36 (62.1%)
**• Female**	22 (37.9%)
**Median Age [y]**	52 (18 – 68)
**MGMT methylation status**	
**• Methylated**	20 (34.5%)
**• Not methylated**	28 (48.3%)
**• Unknown**	10 (17.2%)
**Surgery**	
**• Yes**	47 (81%)
**• No**	11 (19%)
**WHO grade at relapse**	
**• III**	12 (20.7%)
**• IV**	46 (79.3%)
**KPS**	Median 80
**• < 70**	14 (24.1%)
**• ≥ 70**	44 (75.9%)
**Brachytherapy**	
**• Yes**	8 (13.8%)
**• No**	50 (86.2%)

All MG patients (N = 58) had received radiotherapy with median 60 Gy as a postoperative or primary therapy before.

### Time interval

The time interval between first and second irradiation was calculated to be in median 21.4 months, i.e. 642 days (range, 173 – 8112 days). The shortest duration between first and second irradiation was 173 days (5.8 months), but in general, time intervals were intended to be above six months which is seen as a critical time limit for re-irradiation. The duration between first and second irradiation had no influence on post-recurrence survival/progression-free survival (PFS) in univariate analysis (Cox regression, p = 0.57/0.53).

### Treatment parameters

An overview on the treatment parameters is given in Table 2 (for primary and re-irradiation). The EUDs for GTV, PTV and OARs are shown as well as the volume of the GTV (and corresponding spherical radius to derive a comparable 2D-measure). Number of available data sets, maximum (median) values of relevant dose parameters (D_max_, EUD) are shown.

**Table 2:** Treatment parameters

	**RT 1**			**Re-RT**		
**Target structure**	**N**_**1**_	**Median**_**1**_	**Maximum**_**1**_	**N**_**2**_	**Median**_**2**_	**Maximum**_**2**_
**EUD (optic chiasm) [Gy]**	25	40.5	54.3	58	7.4	27.4
**Maximum dose (optic chiasm) [Gy]**	25	49.5	55.9	58	9.8	27.7
**EUD (brainstem) [Gy]**	27	42.0	55.4	58	15.1	43.0
**Maximum dose (brainstem) [Gy]**	29	53.2	61.7	58	22.3	50.1
**EUD (left optic nerve) [Gy]**	26	18.8	50.9	58	2.0	23.1
**Maximum dose (left optic nerve) [Gy]**	26	25.0	55.3	58	2.2	27.8
**EUD (right optic nerve) [Gy]**	23	18.2	53.0	58	3.0	24.5
**Maximum dose (right optic nerve) [Gy]**	24	18.6	55.2	58	3.2	25.2
**EUD (brain) [Gy]**	12	45.0	50.1	52	27.1	32.5
**Mean dose (brain) [Gy]**	12	25.0	40.3	55	12.2	20.2
**Maximum dose (brain) [Gy]**	12	63.4	64.4	55	37.6	50.4
**Volume GTV [Gy]**	21	36.6	423.4	51	33.9	157.9
**Spherical radius GTV [cm]**	21	2.1	4.7	51	2.0	3.4
**EUD**_**GTV **_**[Gy]**	19	60.1	60.7	50	36.1	47.3
**EUD**_**PTV **_**[Gy]**	22	59.3	61.9	50	33.7	43.9

Various parameters on re-irradiation for the patient population are shown.

At primary treatment, the median tumor GTV volume was 36.6 cc (range, 8.0–423.40 cc). If this is converted into a spherical volume, the minimum value of the maximum diameter (=2× radius) of the tumor may be estimated: r = 2.1 cm (median), (range, 1.2–4.6 cm). The median re-treatment GTV volume was 33.9 cc, (range, 1.9–157.9 cc) and the corresponding spherical radius: r = 2.0 cm (median), (range, 0.8– 3.4 cm). GTV volumes were not significantly different comparing primary and re-irradiation (p = 0.14) but PTVs differed significantly (292 cc vs. 120 cc, p < 0.001) which is due to the respective margin concept.

Concerning the optic chiasm, median EUD/maximum dose at primary therapy was 40.5 Gy/49.5 Gy and during re-irradiation 7.4 Gy/9.8 Gy. For the brainstem median EUD/maximum dose at primary therapy was 42.0 Gy/53.2 Gy and during re-irradiation 15.1/22.3 Gy. For the optic nerves (left and right nerve were evaluated separately) median EUD/maximum dose at primary therapy was (left): 18.8 Gy/25.0 Gy (right: 18.2 Gy/18.6 Gy) and (left) 2.0 Gy/2.2 Gy during re-irradiation (right: 3.0 Gy/3.2 Gy).

Concerning the brain, median EUD/mean dose/maximum dose at primary therapy were 45.0 Gy/25.0 Gy/63.4 Gy and during re-irradiation 27.1 Gy/12.2 Gy/37.6 Gy.

For those patients with available data sets and/or DVHs at primary and re-irradiation cumulative EUD values were determined (Table 3). Median cumulative EUD/max dose for the optic chiasm was 48.8 Gy/57.7 Gy, for the brainstem 57.4 Gy/74.9 Gy, for the left optic nerve 22.1 Gy/28 Gy, for the right optic nerve 20.9 Gy/23.3 Gy and for the brain 73.8 Gy/101.5 Gy.

**Table 3:** Cumulative treatment parameters

		**RT 1 + Re-RT**		**Corrected sum**	
**Target structure**	**N**	**Median**	**Maximum**	**Median**	**Maximum**
**EUD (optic chiasm) [Gy]**	25	48.8	76.5	29.0	50.3
**Maximum dose (optic chiasm) [Gy]**	25	57.7	80.3	32.0	53.6
**EUD (brainstem) [Gy]**	27	57.4	75.3	36.0	56.9
**Maximum dose (brainstem) [Gy]**	29	74.9	95.2	46.4	72.7
**EUD (left optic nerve) [Gy]**	26	22.1	60.2	14.2	35.7
**Maximum dose (left optic nerve) [Gy]**	26	28.0	64.7	15.6	38.1
**EUD (right optic nerve) [Gy]**	23	20.9	68.3	12.1	41.8
**Maximum dose (right optic nerve) [Gy]**	24	23.3	79.4	13.3	52.0
**EUD (brain) [Gy]**	10	73.8	77.3	50.8	53.9
**Mean dose (brain) [Gy]**	11	37.6	52.7	23.5	32.6
**Maximum dose (brain) [Gy]**	10	101.5	104.5	69.5	72.8

Various parameters on first and re-irradiation for the patient population are shown.

Employing a recovery factor of 0.5 this resulted in the following median EUDs/maximum doses: 29.0 Gy/32.0 Gy for the optic chiasm, 36.0 Gy/46.4 Gy for the brainstem, 14.2 Gy/15.6 Gy for the left optic nerve, 12.1 Gy/13.3 Gy for the right optic nerve and 50.8 Gy/69.5 Gy for the brain.

### Correlation of tumor volume and survival

In a second step, individual tumor volumes at re-irradiation were compared and correlated with treatment outcome (survival from the beginning of re-irradiation/progression-free survival).

First of all, we calculated spherical volumes for different radii using the well-known formula V = 4/3*π*r^3^ and obtained different volume cut-offs to be tested (concerning the GTV). These values were used to define different treatment groups which were separated by their GTV volume (two patients were excluded from this analysis due to distant and distinct tumoral lesions).

We therefore obtained V(r = 2 cm) = 34 cc (25/26 patients within the respective group, smaller or larger than the defined volume). The comparison revealed a non-significant result: p(r = 2 cm) = 0.63 (survival) and p = 0.62 (PFS).

### Univariate analyses

No significant influence on post-recurrence survival was seen for sex, age, WHO grade at recurrence, previous surgery, MGMT methylation status, time interval between first and second therapy session and radius of the re-treated volume (assuming a spherical mass). Karnofsky performance status (KPS) was a significant prognostic factor with a median survival of 308 days vs. 176 days for patients with KPS < 70 (log-rank p = 0.003), for an overview see Table 4.

**Table 4:** Results of the univariate analysis

**Variable**	**Univariate p-value PRS/PFS/OS**
**Age**	ns / ns / ns
**KPS (< 70, ≥ 70) at re-RT**	0.003 / 0.009 / -
**surgery (yes/no)**	ns / ns / 0.01
**MGMT (meth/not meth)**	ns / ns / ns
**EUD GTV/PTV**	ns / ns / ns
**PTV volume**	ns / ns / -
**WHO grade at relapse (III/IV)**	ns / 0.05 / 0.04
**Sex (male/female)**	ns / ns / ns
**Time interval between first and re-RT**	ns / ns / 0.001

P-values are shown for different treatment factors/characteristics, influence on post-recurrence survival (PRS), post-recurrence progression-free survival (PFS) and overall survival (OS). ns – not significant.

Concerning PFS post re-irradiation, age group, surgery, MGMT (10 cases missing), minimum GTV/PTV dose, spherical GTV radius/volume, time interval between first and re-irradiation and sex were no significant prognostic factors. WHO grade was significantly associated with PFS; median PFS for grade IV tumors was 214 compared to 145 days for grade III tumors, p = 0.05; KPS dependence was again pronounced: enhanced PFS within the better KPS group: 169 vs. 234 days, p = 0.009.

Taking the time of initial radiotherapy as starting point, overall survival time was analyzed on the relevance of possible prognostic factors. Median overall survival for younger patients (<60 years) was 1275 days compared to 879 days (p = 0.09). Sex, minimum GTV/PTV dose, PTV volume, initial volume/corresponding spherical radius did not reach significance. WHO grade had a significant prognostic influence favoring grade III vs. IV: 2607 vs. 976 days median survival (p = 0.04) as well as surgery (p = 0.01) with a benefit of 1545 days vs. 1033 days favoring surgery. TMZ was a significant factor for worse survival (2607 vs. 879 days) which was obviously due to WHO grade III patients who have not been treated with temozolomide as a first-line schedule (p = 0.003) and with a small number of long-term glioblastoma (GBM) survivors who have not been treated with TMZ initially (selection bias). MGMT methylation status did not reach significance, either (p = 0.12); but MGMT methylated patients survived 2231 days vs. 1275 days. Concerning the EUD to the GTV no significant influence on OS could be detected, HR 0.84 (95%CI 0.58; 1.20), p = 0.33; the influence of the EUD to the PTV was even less pronounced, HR 1.0 (95%CI 0.9; 1.1), p = 0.72. The time interval (median 573 days, range 173–8112 days) between first and second irradiation was as expected a highly significant prognostic factor (p < 0.001) as longer intervals indicate a longer treatment history and thus regularly enhanced survival.

## Discussion

As recently shown in several studies, re-irradiation is a safe and feasible therapy option for recurrent MG. Modern and highly conformal treatment approaches allow brain re-irradiation for palliative treatments with low to acceptable probability of radiation necrosis.

Aim of this retrospective study was to determine different treatment parameters of re-irradiation such as maximum dose, mean dose, treated volume, EUD as well as their cumulative estimates and correlate these parameters with survival. A focus was set on EUD as a well-defined parameter being objectively capable of inhomogeneous dose distributions having an advantage compared to peak dose estimates.

In this regard, this study provides for the first time a comprehensive set of treatment parameters including EUD values for primary and re-irradiation of MG. Median cumulative EUD to the optic chiasm was 48.8 Gy (range, 2.5–76.5 Gy), 57.4 Gy (range, 2.7–75.3 Gy) to the brainstem, 20.9/22.1 Gy (range, 0.0–68.3 Gy) to the right/left optic nerve and 73.8 Gy (range, 64.9–77.3 Gy) to the brain. No correlation between treated volume and survival was seen.

Due to their integral definition, EUDs are much more informative compared to e.g. the maximum doses. Conservative estimates for the actual cumulative EUD were derived as sum of previous EUD and EUD of re-irradiation [30]; considering maximum values, the estimation would be too conservative as hotspots of primary and re-irradiation are regularly in different areas of the brain, for mean values this estimate would be exact. In many cases, there is no possibility to co-register these two datasets and to derive a cumulative dose map; so EUDs are of even higher importance in future.

There was no relevant toxicity in regard to radiation necrosis in this patient cohort making this treatment a reasonable and effective approach [25]. Imaging and histo-pathology revealed at maximum three cases with changes compatible with radiation necroses whereas diagnosis mainly relied on MRI suspected lesions rather than clinical deterioration. Rates of leukoencephalopathy ≥ G3 were negligible and ≥ G2 very low. Time interval between first and second irradiation was regularly above 6 months in our study and did not correlate with post-recurrence survival.

Our results are slightly different from previous studies especially in regard to the meaning of tumor volume as a prognostic parameter [11,31], but this might be due to the use of bevacizumab [32] which was not applied in other studies. Concerning the surprising results for PFS/post-recurrence survival according to WHO grade, one has to state the retrospective nature of this analysis and another reason and potential bias is the fact that some of the grade III tumors could have been GBMs at recurrence if they were only diagnosed by RANO criteria.

With median cumulative EUD to the brain of 73.8 Gy and an acceptable toxicity, our results are in the range of Mayer et al. who showed that radiation-induced normal brain tissue necrosis occurs at normalized total doses >100 Gy (cumulative normalized total dose, NTD_cumulative_). The applied re-irradiation dose and NTD_cumulative_ increase with a change in irradiation technique from conventional to radiosurgery re-treatment [33]. Unfortunately, no defined retreatment tolerance doses for brainstem, optic chiasm or nerves are currently available. In our practice adapted from the re-treatment data of animals published by Ang et al. [19] we assume half repair of the damaged neural tissue after one year. Then the potentially remaining dose according to known tolerance doses is calculated [34-37]. Thus, the cumulative doses were mainly below existing thresholds.

In the present analysis many interesting therapeutic parameters are derived but nevertheless, it has several shortcomings.

First of all, many treatment plans of the primary RT session were not available (in electronical form) which limits EUD analysis – in several cases hardcopies were existing but several OARs had not been contoured.

Furthermore, a longer follow-up and a larger case number are needed to derive a meaningful NTCP model correlating normal tissue dose and toxicity. In the present analysis, for EUD calculation certain model parameters were assumed as no exact values are known and data in the literature is sparse on this topic.

Another uncertainty was introduced as cumulative dose parameters were calculated using digitalized previous planning system data (of treatment systems different from OTP) each employing algorithms with different calculation accuracies.

## Competing interests

The authors declare that they have no competing interests.

## Authors’ contributions

UG, CB & MN planned, coordinated and performed the study. IK collected and analyzed the data. PL provided information on physical treatment planning. MS provided assistance on EUD theory as well as EUD calculation. MN, MS, CB, SBN & UG prepared the manuscript. All authors read and approved the final manuscript.
